# *SAXS4COLL*: an integrated software tool for analysing fibrous collagen-based tissues

**DOI:** 10.1107/S1600576717007877

**Published:** 2017-07-07

**Authors:** Ahmed Abass, James S. Bell, Martin T. Spang, Sally Hayes, Keith M. Meek, Craig Boote

**Affiliations:** a Structural Biophysics Group, School of Optometry and Vision Science, Cardiff University, Maindy Road, Cardiff CF24 4HQ, UK

**Keywords:** small-angle X-ray scattering, fibrillar collagen, data reduction, data processing

## Abstract

This article provides an overview of a new integrated software tool for reduction and analysis of small-angle X-ray scattering (SAXS) data from fibrous collagen tissues, with some wider applicability to other cylindrically symmetric scattering systems. *SAXS4COLL* combines interactive features for data pre-processing, bespoke background subtraction, semi-automated peak detection and calibration. Both equatorial and meridional SAXS peak parameters can be measured, and the former can be deconstructed into cylinder and lattice contributions. Finally, the software combines functionality for determination of collagen spatial order parameters with a rudimentary orientation plot capability.

## Abstract 2

*SAXS4COLL* is an interactive computer program for reduction and analysis of small-angle X-ray scattering data from fibrous collagen tissues, combining data reduction, bespoke background subtraction, semi-automated peak detection and calibration.

## 1. Introduction   

**Figure 1:**
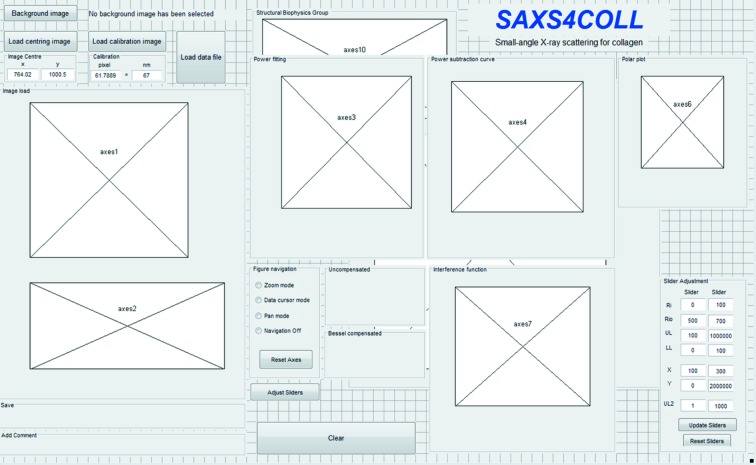
Layout of the *SAXS4COLL* GUI, illustrating the positions of the main processing and display windows, including image display windows (axes 1 and 2), background fitting and display windows (axes 3 and 4), the fibre orientation plot window (axes 6), and the collagen fibril lattice function display window (axes 7).

With continual advances in synchrotron power and beamline technology, the use of small-angle X-ray scattering (SAXS) techniques to study biomolecular structures has increased significantly in the past few decades (Jacques & Trewhella, 2010 ▸). The widening application of synchrotron-based SAXS methods has driven efforts to produce computing applications capable of analysing increasingly large SAXS datasets more efficiently (Jacques & Trewhella, 2010 ▸). One area in which these benefits have been felt is in the study of noncrystalline and semi-crystalline fibrous materials, including synthetic polymers, DNA, muscle and collagen-based connective tissues. Between 1992 and 2005 a productive partnership between synchrotron and academic institution personnel [Collaborative Computing Project (CCP)13; Squire *et al.*, 2003 ▸] led to the development of a suite of software for use by the small-angle scattering and fibre diffraction communities. Most notably, the generic CCP13 routine *FibreFix* (Rajkumar *et al.*, 2007 ▸) was developed to allow semi-automatic determination of structural parameters from noncrystalline diffraction patterns. *FibreFix* is an integrated software suite for Windows and Linux operating systems which features an interactive graphical user interface (GUI) to select, process and present data. Further, more specialized software has also been developed under CCP13. These include *HELIX* (Squire & Knupp, 2004 ▸) for simulation of helical molecules and *MusLABEL* (Squire & Knupp, 2004 ▸) for modelling striated muscle. However, currently there exists no integrated software tool aimed specifically for the analysis of SAXS patterns from collagen-based tissues. This paper represents a new interactive SAXS data analysis program originally produced for the investigation of corneal collagen structure and suitable for processing SAXS data from any fibrous collagen specimen. The presented *SAXS4COLL* software is a GUI-based system for pre-processing of fibrous collagen SAXS patterns, fitting bespoke power-law background functions to the integrated scattered signal, identifying and calibrating the equatorial (interference function and cylinder transform) and meridional (axial D period) peaks, and semi-quantitatively determining preferential collagen orientation. The program supports semi-automated batch processing to allow rapid loading and analysis of similar files, such that background and cylinder transform parameters can be retained or quickly modified. This program was developed in MATLAB R2016b (The MathWorks Inc., Natick, MA, USA) as a GUI application. An overview of the GUI layout is presented in Fig. 1 ▸.

## 2. Data pre-processing   

### 2.1. Input file format   

Input files should be converted to a standard high-resolution image file format compatible with the imread.m function in MATLAB (accepted formats include TIFF, JPEG or PNG) for processing in the *SAXS4COLL* software.

### 2.2. Pattern centring   

**Figure 2:**
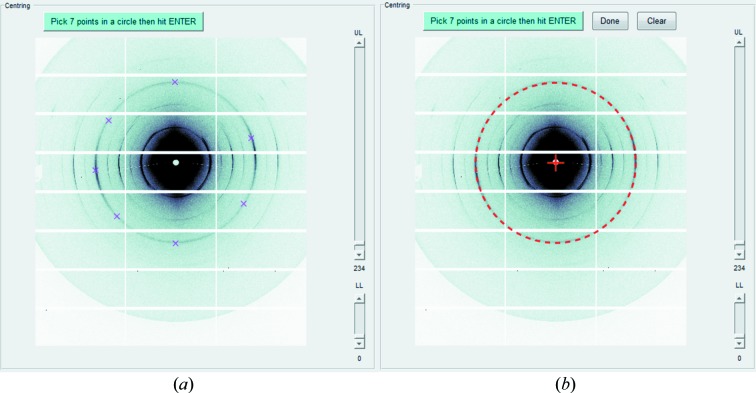
Pattern centring. (*a*) Selection of seven points (crosses) on a chosen calibrant diffraction ring (in this case from tendon collagen). (*b*) Calculated centre (red cross). The dashed red circle represents the best fit to the selected points.

*SAXS4COLL* enables semi-automated centring of the SAXS pattern. A pattern with circular peaks is loaded, for example from a calibrant such as tendon collagen, silver behenate, silicon *etc*. The user is then instructed to select seven points around the circumference of one of the calibrant diffraction rings (Fig. 2 ▸ *a*). The software uses the MATLAB routine *Circle fit* (http://www.mathworks.com/matlabcentral/fileexchange/5557-circle-fit) to automatically calculate the geometric centre of the selected circle and records it as the pattern centre/straight-through beam position (Fig. 2 ▸ *b*). *SAXS4COLL* allows the user to repeat the centring process, if necessary.

### 2.3. Negative data correction   

Where detector calibration leads to the raw SAXS patterns containing negative intensity data, *SAXS4COLL* makes an automatic correction in which the minimum value of the intensity matrix is subtracted from the whole matrix in order to shift magnitudes above zero.

### 2.4. Background image subtraction   

*SAXS4COLL* gives the user the option of setting a certain SAXS image as a background image. This is used to remove/reduce background scatter such as flare from the beamstop and diffuse scatter from the specimen cell. If the user does not identify a background image, the software sets the background matrix to zeros.

### 2.5. Removal of outlier points   

**Figure 3:**
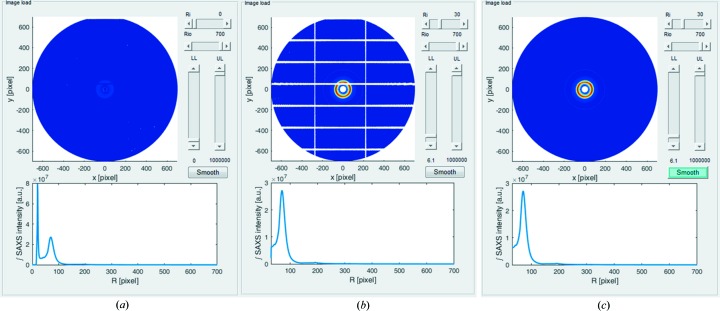
Removal of outliers and recovery of undetected areas of unprocessed patterns. (*a*) Original unprocessed image. (*b*) Adjusting slider limits; the lower limit (LL) threshold has been set to 6.1 such that undetected data (in this case from detector gaps) appear as white areas. (*c*) The same pattern after compensation. The SAXS azimuthally integrated signal is shown under each pattern.

Outlier points, such as beamstop flare from the main X-ray beam, may be removed interactively and considered as missing points, or not a number (NaN). In the GUI, moveable upper limit (UL) and lower limit (LL) sliders enable the user to exclude any outlier element in the X-ray pattern matrix and treat it as a missing data point. A radial limit range for the azimuth integration is selected using the limit sliders, lower radial limit (Ri) and upper radial limit (Rio), as shown in Fig. 3 ▸. A before and after comparison of the use of the limit sliders is shown in Figs. 3 ▸(*a*) and 3 ▸(*b*). The pattern colour scale is automatically updated accordingly.

### 2.6. Compensation of missing data   

Owing to the modular nature of some X-ray detectors, such as the Pilatus range, data may be missing in the gaps between the detector modules (Fig. 3 ▸ *b*). Assuming negligible out-of-plane effects, the SAXS pattern from a collagenous tissue has rotational symmetry of order 2 about the scattering centre, and this symmetry can be used to populate regions of lost data. By pressing the ‘Smooth’ button, the software considers each white pixel and looks for a valid data point at the symmetrically opposite point of the pattern to populate it with. Circumferential interpolation is then used to populate any remaining lost data points, which can be minimized through careful selection of the centre point at the start of a SAXS experiment. Once all the white pixels have been populated with data, the reconstructed SAXS data pattern is displayed (Fig. 3 ▸ *c*).

## 3. Calibration   

**Figure 4:**
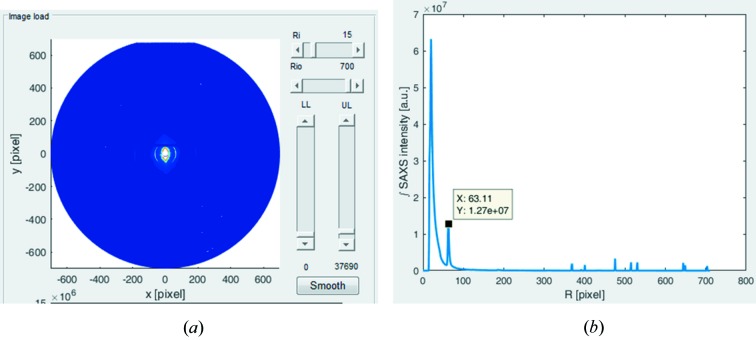
Interactive calibration using rat-tail tendon collagen. (*a*) Calibrant SAXS pattern displayed in the *SAXS4COLL* GUI. (*b*) Radial SAXS signal display, showing manual selection of the collagen meridional first-order peak position to be used for calibration.

The loading of a calibration file, such as a SAXS pattern from hydrated rat-tail tendon, results in a display of the raw pattern alongside the azimuthally integrated radial SAXS profile (Fig. 4 ▸). The user then selects an appropriate calibration peak, for example the first-order meridional peak from tendon collagen, by clicking on the integrated SAXS intensity plot (Fig. 4 ▸ *b*). The pixel value of the calibration peak, as displayed by the software (Fig. 4 ▸ *b*), is then entered into the calibration input box alongside the corresponding period in nanometres, for example 67 nm for the collagen first order (Fig. 4 ▸ *a*). These values will be stored by *SAXS4COLL* and subsequently used to automatically calibrate the specimen collagen peak positions.

## 4. Interactive background fitting   

**Figure 5:**
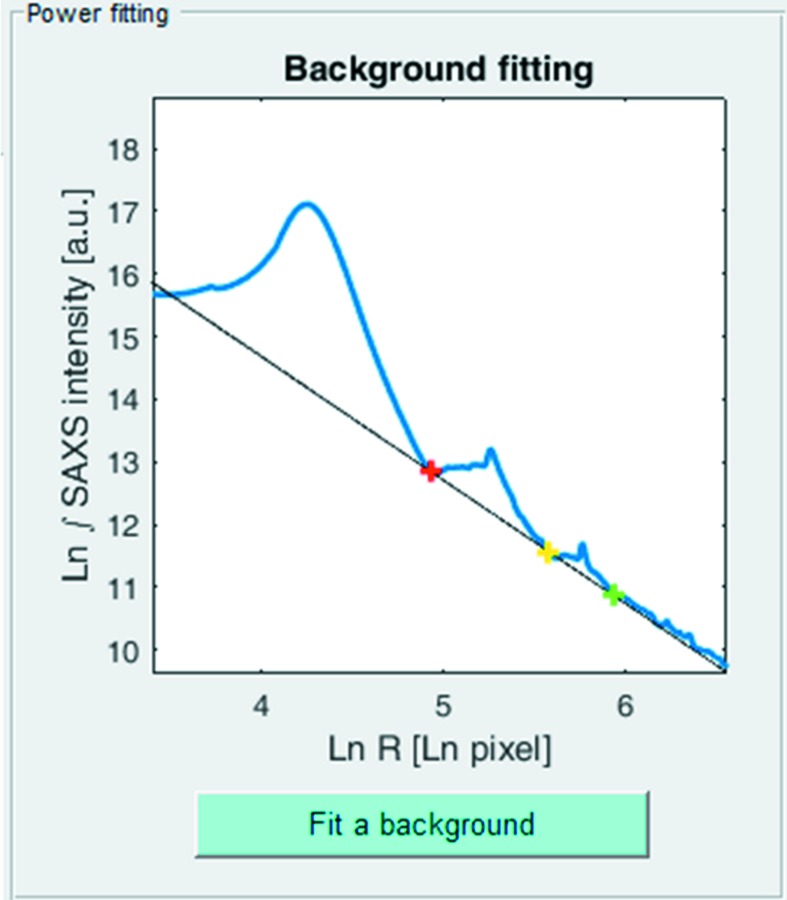
Power function background fitting. The azimuthally integrated scattering signal (blue) is fitted with a power-law background function (black). User-selected fitting points are shown as red, yellow and green crosses.

Residual scatter from the specimen cell and non-collagen components of the specimen can be interactively removed by fitting a background function to the pre-processed data. The software invites the user to specify three points on the azimuthally integrated SAXS signal, presented as a double natural log plot (Fig. 5 ▸). The program then fits a straight-line background function to the chosen points, which equates to the general power function in linear space
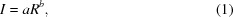
where *I* is the integrated SAXS intensity, *R* is the radial pixel position, and *a* and *b* are constants. An adjustable zoom option facilitates accurate fitting of the background function and the program allows repeated fittings.

## 5. Collagen peak detection and fitting   

### 5.1. Calculation of structure parameters   

**Figure 6:**
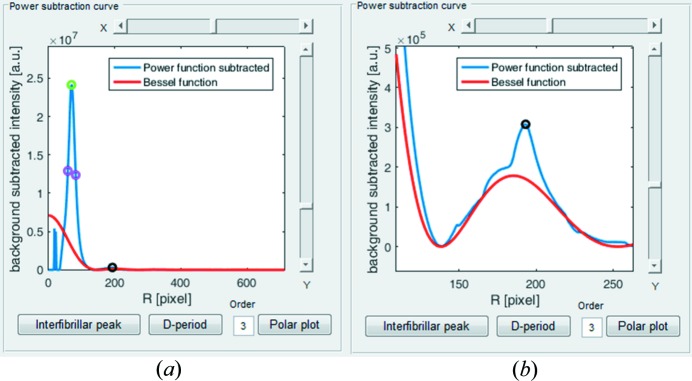
Collagen peak detection and fibril transform (Bessel function) fitting. (*a*) Power-function-subtracted data with fitted Bessel function curve and the interfibrillar peak (green circle) and third-order meridional peak (black circle) detected. Magenta circles show the detected HHW limits. (*b*) Expanded view of the Bessel function fitting.

*SAXS4COLL* enables the identification of a meridional peak from a plot of the background-subtracted, azimuthally integrated SAXS signal, and allows the user to input the order of the reflection (*e.g.* from the collagen axial D period). The user is required to click near the peak and the software will then automatically find the nearest maximum. In some patterns there may exist equatorial reflections arising from lateral order in the collagen fibril spatial organization. The program gives the user the option of manually identifying the collagen equatorial interference function peak, referred to as the interfibrillar peak in the GUI, from which the average Bragg collagen fibril separation distance is calculated (Fig. 6 ▸ *a*). Again the program will detect the closest peak to the selected point. The software then requires the user to manually adjust the fit of the equatorial fibril (cylinder) transform, which takes the form of a Bessel function of the first kind (see §5.2), using the sliders to the right of the plot (Fig. 6 ▸ *b*). The position of the first subsidiary Bessel peak is used to calculate the average collagen fibril diameter (Meek & Quantock, 2001 ▸). The software automatically calibrates the positions of the meridional peak, the Bessel function peak and the interfibrillar peak to determine, respectively, the D period, the fibril diameter and, to a first approximation, the Bragg fibril separation and the relative spatial order of collagen (see §5.2). The order parameter (in a.u.) is computed from the interfibrillar peak height divided by the half height width (HHW).

### 5.2. **Refinement of the interference function**   

**Figure 7:**
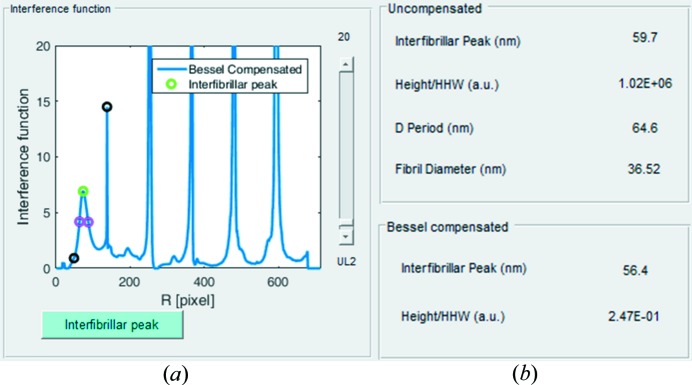
Division of the cylinder transform to obtain the Bessel-compensated interference function. (*a*) New interfibrillar peak (green circle) selection from Bessel-compensated interference function. Black circles show the search region of the interfibrillar peak and magenta circles show detected HHW limits. (*b*) The results table, displaying collagen structure parameters before (top panel) and after (bottom panel) Bessel compensation. The last line of the top panel shows the fibril diameter calculated from the fitted Bessel function used for compensation.

The equatorial integrated SAXS pattern from a fibrous collagen specimen, *I*(*K*), is the product of the ‘interference function’ from the lattice, *G*(*K*), and the scattered amplitude of a single collagen cylinder – often termed the ‘fibril transform’ or ‘cylinder transform’, *F* ^2^:
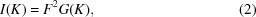
where *K* is the scattering vector magnitude (Meek & Quantock, 2001 ▸). If we assume that the collagen cylinder length is infinitely longer than its radius, and that the electron density along its length is uniform, then the scatter amplitude can be represented as
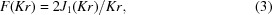
where *r* is the cylinder radius and *J*_1_ is a Bessel function of the first kind (Oster & Riley, 1952 ▸). The intensity profile of the cylinder transform, *F* ^2^(*Kr*), has maxima when *Kr* = 0, 5.14, 8.42 *etc*. Hence, in cases where the fibril sizes are sufficiently uniform to generate a well defined, unimodal cylinder transform, the software allows division of the Bessel function from the background-subtracted data to yield a Bessel-compensated interference function (Fig. 7 ▸ *a*) and allows the user to detect the shifted interfibrillar peak from this compensated function (Fig. 7 ▸ *a*). Bessel-compensated interfibrillar spacing and height/HHW order parameters are calculated and displayed alongside the non-Bessel-compensated values (Fig. 7 ▸ *b*).

## 6. Collagen orientation   

**Figure 8:**
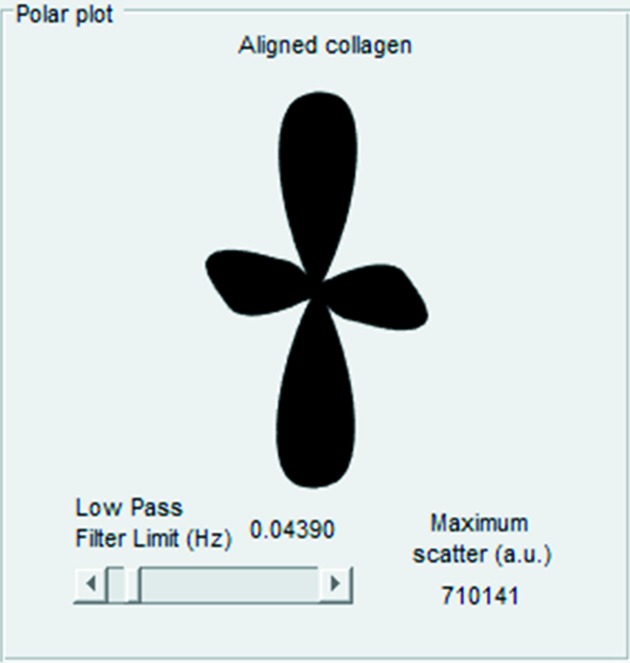
Collagen orientation polar plot. The aligned collagen scatter (from fibrils preferentially oriented over and above any underlying randomly oriented collagen) is represented as a polar vector plot. The length of a vector from the plot centre to edge in any direction is proportional to the relative number of collagen fibrils preferentially oriented at that angle. In this example the orientation distribution of the fibrils is approximately orthogonal bimodal.

*SAXS4COLL* is able to produce polar vector plots showing the predominant orientation of collagen in each SAXS pattern. Collagen fibril orientation data from X-ray scattering patterns may be useful for biomechanical interpretation of the tissue and, for example, have been shown to be highly amenable for application in finite element analyses (Whitford *et al.*, 2015 ▸; Coudrillier *et al.*, 2015 ▸). The user is required to identify the radial positions of the inner and outer integration limits by clicking each side of the peak of interest in the power subtraction curve. The software then displays the orientation polar plot (based on the azimuthal distribution of radially integrated intensity) automatically. This flexibility allows the orientation to be calculated from either the equatorial (interfibrillar peak) or meridional SAXS signals. The software has an adjustable zero-phase low-pass filter to smooth the polar plot shape. The variable plotted is the preferentially aligned collagen, defined as the total azimuthal scatter after background subtraction minus the minimum azimuthal (*i.e.* isotropic) scatter (Meek & Boote, 2009 ▸) (Fig. 8 ▸). The polar plot is saved automatically as a TIFF file, while the data from the plot (aligned scatter *versus* azimuth angle) are saved to an Excel spreadsheet.

## 7. Batch processing   

By default, once the first data file is loaded, *SAXS4COLL* enters semi-automated batch processing mode. Consecutive images may be loaded with a single click and processed automatically with the same background and cylinder transform parameters applied. Alternatively the processing parameters can be modified as desired. The output polar plot and structure parameter data from a batch are saved within common Excel spreadsheets unless directed otherwise by the user.

## 8. Program availabilty   

This software is available as a stand-alone executable which does not require MATLAB to be installed on the user’s computer. However MATLAB will be required to run the source code. Microsoft Excel is required to view certain saved data files. The *SAXS4COLL* software has been developed for research purposes with no commercial interest. Users can obtain the program at no cost by downloading the stand-alone executable and MATLAB source code/supporting files at the following addresses:

Executable:

Doi: https://doi.org/10.6084/m9.figshare.4775743

Web link: https://figshare.com/s/7be18c69b8b0bd113db7

Code:

Doi: https://doi.org/10.6084/m9.figshare.4776478

Web link: https://figshare.com/s/94d1afd980b785b99d69

User Guide:

Doi: https://doi.org/10.6084/m9.figshare.4776484

Web link: https://figshare.com/s/9c7c7e5873610d8c6d1d

## 9. Conclusion   

*SAXS4COLL* was designed to satisfy the requirement for rapid automated data reduction and analysis of increasingly large collagen SAXS datasets, while retaining sufficient user interaction and flexibility to cater for variance in data type and quality across specimen types and different SAXS beamlines. The software displays all processing stages and gives the user full control to accept or repeat and adjust parameters at any stage until the user is satisfied with the result. The software produces an organized Microsoft Excel spreadsheet with a record of the data processing stages and results.

*SAXS4COLL* combines interactive features for data pre-processing, bespoke background subtraction, semi-automated peak detection and calibration. Both equatorial and meridional SAXS peak parameters can be measured, and the former can be deconstructed into cylinder and lattice contributions. Finally, the software combines functionality for determination of collagen spatial order parameters with a rudimentary orientation plot capability. While complete automation of the *SAXS4COLL* processing is not practical given the amount of user judgment required in analysis, the program maximizes efficiency by supporting a quick-load feature and semi-automated batch analysis. Directories of related files can be rapidly loaded with one click and the original background/cylinder transform fitting parameters either retained or quickly modified. In addition to collagen-based tissues, *SAXS4COLL* could be adapted for use in other cylindrically symmetric scattering systems such as lamellar polymers (*e.g.* latex, silks *etc*.). The MATLAB source code is available to download for users who require modification of the program functionality (*e.g.* bespoke background functions) to cater for more diverse datasets.
